# Time-resolved dynamics of granular matter by random laser emission

**DOI:** 10.1038/srep02251

**Published:** 2013-07-22

**Authors:** Viola Folli, Neda Ghofraniha, Andrea Puglisi, Luca Leuzzi, Claudio Conti

**Affiliations:** 1 Department of Physics, University Sapienza, Piazzale Aldo Moro, 5, 00185, Rome (IT); 2 ISC-CNR, UOS Roma Sapienza, Piazzale Aldo Moro 5, 00185, Rome (IT); 3 IPCF-CNR, UOS Roma Kerberos, Univ. Sapienza, Piazzale Aldo Moro 5, 00185, Rome (IT)

## Abstract

Because of the huge commercial importance of granular systems, the second-most used material in industry after water, intersecting the industry in multiple trades, like pharmacy and agriculture, fundamental research on grain-like materials has received an increasing amount of attention in the last decades. In photonics, the applications of granular materials have been only marginally investigated. We report the first phase-diagram of a granular as obtained by laser emission. The dynamics of vertically-oscillated granular in a liquid solution in a three-dimensional container is investigated by employing its random laser emission. The granular motion is function of the frequency and amplitude of the mechanical solicitation, we show how the laser emission allows to distinguish two phases in the granular and analyze its spectral distribution. This constitutes a fundamental step in the field of granulars and gives a clear evidence of the possible control on light-matter interaction achievable in grain-like system.

Vertically shaken granular systems exhibit a wide ensemble of different phenomenological behaviors. Due to their peculiar structural conformation, granular materials^1^,^2^ are described by a complex phase diagram with fluid-like^3^,^4^,^5^, solid-like and liquid-like areas^6^,^7^,^8^, depending on the furnished mechanical energy. Many of the experimental studies on granular materials uses digital photography, and the analysis is mainly limited to the external layers of the sample. Various experimental techniques, able to analyze the internal behavior of a three-dimensional granular system, have been considered to overcome the opacity of the beads and give information on the internal arrangements of the grains, as for example diffusing-wave spectroscopy (DWS)^9^,^10^,^11^, magnetic resonance imaging (MRI)^12^, X-Ray microtomography^13^, and positron emission particle tracking (PEPT)^14^. These techniques analyze the movement of grains and give information about their density or related functions.

In a previous work^15^, we have shown that a granular material embedded in a light amplifying medium can emit laser radiation depending on the configuration of the beads. Here, we employ this light emission to investigate the granular phases in time resolved experiments. In particular, concerning with the diffusive-wave spectroscopy method, our experiment gives comparable information with respect to that obtainable with DWS, that is a well-developed and available technique. However, our method can be applied in the studying of temporal coherent properties of granular matter in absorbing media. In fact, in dealing with active and absorbing materials, the phenomenon of absorption dramatically reduces the diffusive-wave signal due to multiply scattered light and nullify the contribution of the longer light paths to the autocorrelation function. Herein, exploiting the absorbing properties of the active medium that surrounds the granular beads, we magnify its response by pumping the sample with a laser source that is resonant with the used dye. In this way, the motion of light emitting granulars can be studied by their emitted laser radiation and the corresponding temporal autocorrelation function. The usage of laser emission to investigate granular dynamics, presented here, represents a non-invasive and new method that provides a statistical description of the collective dynamical behavior of agglomerations of macroscopic particles in absorbing media.

At variance with previous work^15^, we consider a completely different system, made by glass beads instead of metallic, and a new aspect. Here, the temporal variation of laser spectra from oscillated granular is followed dynamically to extrapolate information on the granular phase and we find that the spectral emission is determined by the beads spatial configuration through some non-trivial internal correlations. These are not accessible by means of other techniques and, in some cases, display remarkably oscillating collective modes.

With respect to standard random lasers (RL)^16^,^17^, we stress that in “granular” lasers, an agglomeration of macroscopic particles in liquid active media, gravity plays a crucial role in affecting the laser emission spectra and represents a new and fascinating degree of freedom, which jointly with the external mechanical solicitation, allows to tune the laser system above and below its threshold. So, we have created a new kind of laser system, a “granular” laser, that has a tunable emission spectrum. In fact, depending on the externally furnished mechanical energy, very many states of the granular are explored and drive the laser emission. In these respects, the dynamics is a fundamental issue in addressing the complexity of the system, as during the evolution the many possible states are explored.

In our opinion, this work could be the starting point to use random laser emission as an effective spectroscopic tool beyond density measurements to investigate internal collective dynamical modes of shaken and gravity affected three-dimensional granular systems in liquid solution.

## Results

### Experimental setup

**Figure 1:**
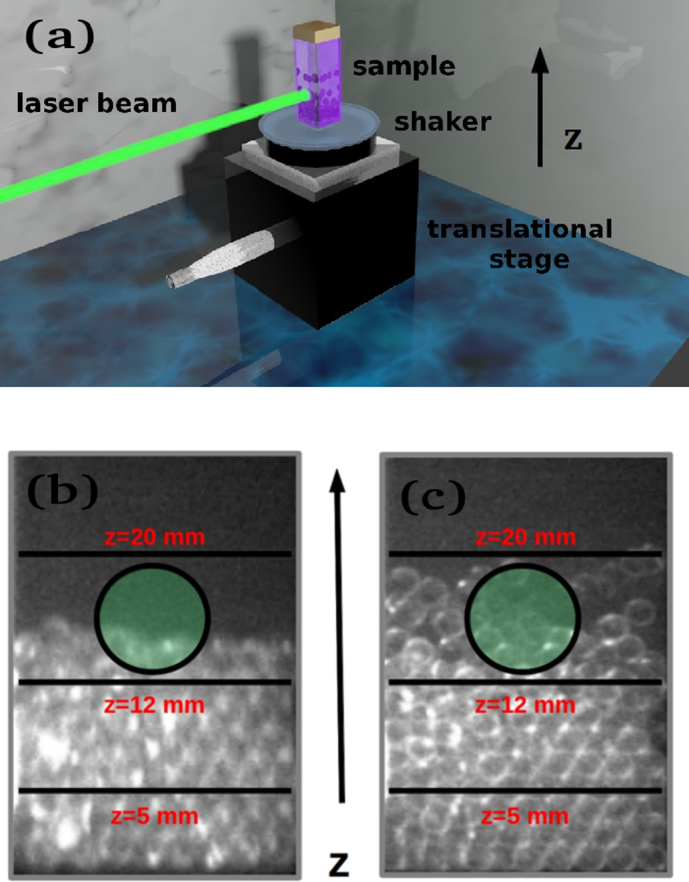
(a) Sketch of the experiment, (b) grain distribution when the bottom plate oscillates with a displacement *a* smaller than the bead diameter *D*: 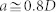; (c) as in (b) with 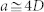, note the enhanced grain displacements.

In the experiment, the sample consists of 1 mm diameter spherical glass grains dispersed in a liquid Rhodamine B solution. It is placed on a shaker that can vertically vibrate at frequency *f*. The vertical displacement (*z*-direction) *a* of the vibrating plane is calibrated by an accelerometer. The structure is arranged on a motorized vertical translational stage (maximum travel of 25 mm). A high energy pump beam is injected on the sample at three different vertical position *z*, fixed when the sample is at rest (*a* = 0). A sketch of the experimental setup is shown in Fig. 1 panel (a), details are found in Methods.

Varying the vibrational parameters and the vertical position allows to study the light-grain matter interaction at several structural arrangements of the grains. Our aim is to use the fast time-scale of emitted light to investigate the much slower dynamics of such structural arrangements. Fig. 1, panels (b) and (c), show the snapshots of the granular configuration in two dynamical regimes, with the pump spot size indicated. Well-known control parameters, namely the normalized shaking acceleration Γ = *aω*^2^/*g* (with *ω* = 2*πf* and *g* = 9.81 *m*/*s*^2^), the shaking strength *S* = *a*^2^*ω*^2^/*gD* (with *D* the bead diameter), and the layers number are adopted to explore the various phases^18^,^19^,^20^,^21^,^22^,^23^,^24^. In this work, we retrieve a phase-diagram by varying the driven frequency *f* and the shaking acceleration Γ.

### Laser emission

We first determine the vertical position *z* of the laser beam with respect to the bottom of the cuvette at which the interaction with the light gets optimized, this corresponds to the optimal grain density for the random laser action^15^. We send a sinusoidal signal with *f* = 70 Hz to impress to the granular an oscillation *a* ~ 1.5*D*, (Γ = 30). In this way a dynamical regime with a relevant grain motion is reached. We fix three different heights *z*, at the bottom (*z* = 5 mm), at the edge (*z* = 12 mm) and at the top region (*z* = 20 mm) of the granular. Note that the vertical position *z* is taken with respect to the position of the base of the cuvette at rest, the three chosen positions are separated by the pump spot size (6 mm).

**Figure 2:**
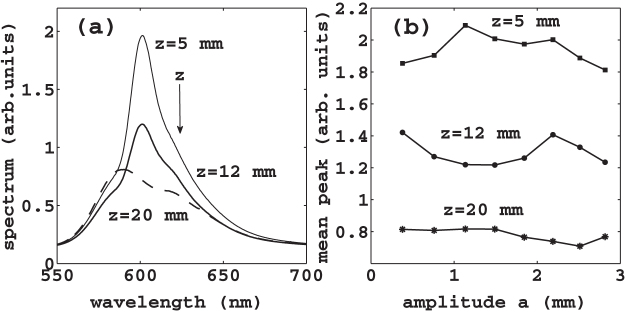
(a) Averaged spectra of shaken granular lasers for exposition time of 0.5 s on 80 accumulations. The input pump energy is 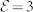 mJ above the random laser threshold, the amplitude and the frequency of the shaking signal are *a* = 1.49 mm and *f* = 70 Hz (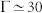). Continuous thin line gives the laser emission at the bottom layers of granular *z* = 5 mm. As the laser spot is moved from the bottom to the top of the container, the laser spectrum becomes less peaked (continuous thick line at *z* = 12 mm and dashed line at *z* = 20 mm); (b) average spectral peak over 80 accumulations versus shaker oscillation amplitude for the three different vertical positions, *z* = 5 mm (squares), *z* = 12 mm (dots) and *z* = 20 mm (asterisks).

**Figure 3:**
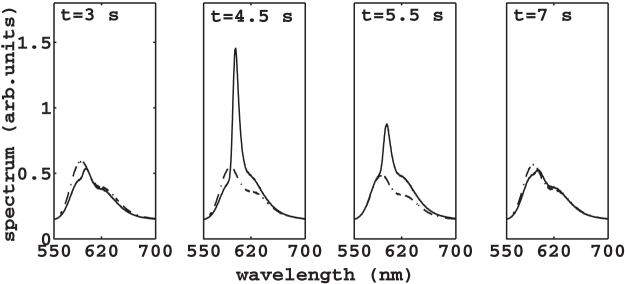
Five-shots spectra in time, taken at *z* = 5 mm (continuous line) and *z* = 20 mm (dashed line). (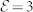 mJ, *a* = 4.96 mm, *f* = 70 Hz, *z* = 5 mm).

**Figure 4:**
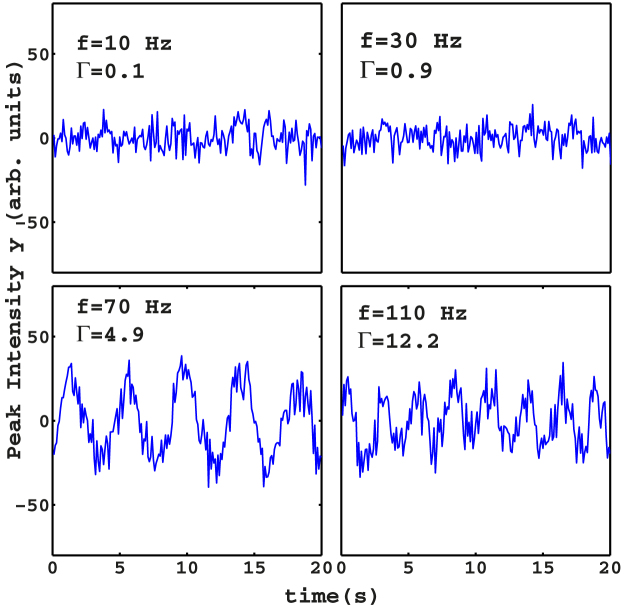
Peak of one-shot laser emission spectra as a function of time for different frequencies, *a* = 0.25. In each plot, we report the corresponding value of *f* and Γ.

**Figure 5:**
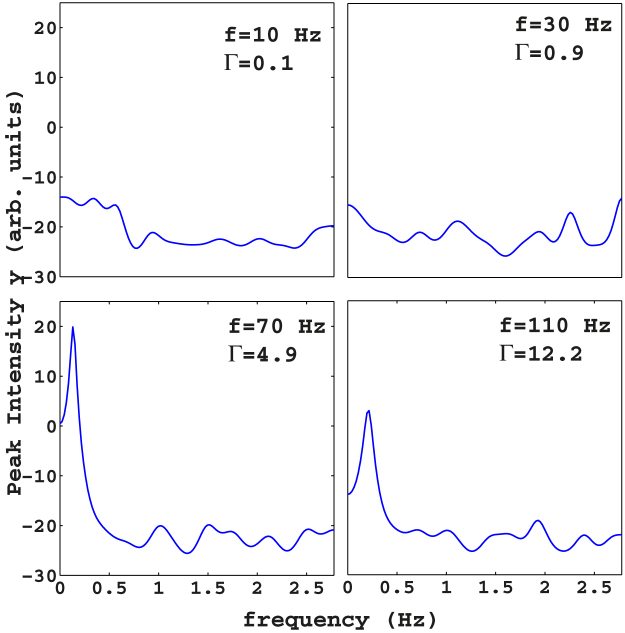
Fourier-domain analysis of the temporal signals in Fig. 4, with shaking frequency *f* and Γ indicated.

**Figure 6:**
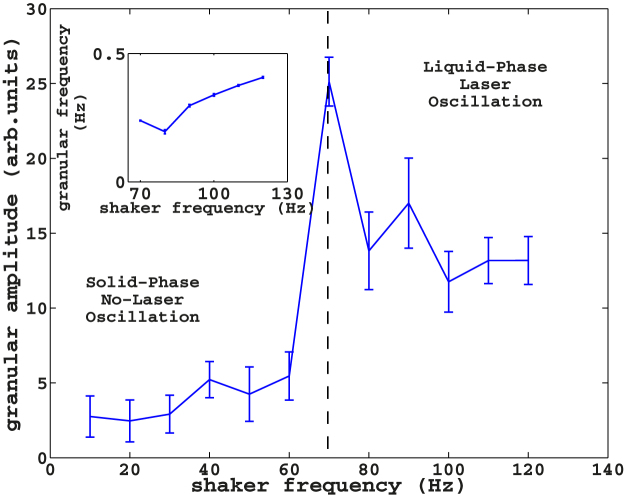
Fitting amplitude of the normalized signals *η* as a function of the frequency. In the inset, it is reported the frequencies of the dynamical evolution of the laser spectra in the oscillating-phase (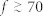).

The averaged spectra in Fig. 2, panel (a), show the laser is more efficient at *z* = 5 mm (continuous thin line). For larger *z*, the laser efficiency is reduced (continuous thick line at *z* = 12 mm and dashed line at *z* = 20 mm) because the granular density decreases with *z* and the laser cavity becomes more lossy as *z* is increased (reduced scattering strength). Fig. 2(b) shows the averaged spectral peak as a function of the shaker displacement; the peak is more pronounced at the bottom region of the cuvette. As shown in^15^, the laser emission of granular shaken laser depends on the furnished mechanical energy and is determined by the specific instantaneous granular configuration. Here, we investigate the granular collective temporal evolution by following the dynamics of its laser emission spectra. We start addressing the time dependence of the granular spectra in Fig. 3. The reported spectra are averaged over five shots (CCD exposure time 0.5 s) and are taken after *t* = 3, 4.5, 5.5 and 7 s from an arbitrary time at two different heights. The spectra are substantially distinct due to a significant variation of the mean grain density. We find that the spectra evolve periodically, and Fig. 3 (a), (b), (c) and (d) correspond to an oscillation period. At first glance, the occurrence of this oscillation is unexpected, indeed the time-scale of the laser emission is much faster than the grain motion, and should be related to the average density that is constant. If the laser emission oscillates, it must sense higher order structural properties. To show evidence that the time dynamics of laser emission allows to discriminate the dynamical evolution of granular matter and it is able to reveal a collective motion underlying the observed density modulation, we proceed as follows: we fix *z* = 5 mm, corresponding to maximal spectral variation (Fig. 3), we choose the pump energy at 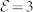 mJ above the random laser threshold, we fix the oscillation amplitude at *a* = 0.25 mm and vary the frequency *f*. During the vibrations of the grains, we collect 200 laser spectra with exposition time 0.1 second (corresponding to a time interval of 36 seconds time interval because of the readout time *t_Rout_* = 0.08 s of the CCD camera) for each measurement. We analyze the experimental results by plotting the peak of each consecutive spectrum as a function of time. This analysis is repeated by making a frequency *f* scan from 10 to 120 Hz with a step of 10 Hz (Fig. 4) in order to explore a wide range of Γ, namely 0.1 ≤ Γ ≤ 14.5. We observe that when the shaker plate is subject to a shaking acceleration approximately less than 5, the whole granular barely moves; correspondingly, the dynamical evolution of the laser spectra is negligible, as the mean bead density is constant with time. When the peak vibration amplitude Γ is increased, the mechanical solicitation on the granular overcomes the gravitational force, the capillary forces between grains and liquid, and the constraints imposed by the sides of the cuvette. In such a regime, the granular is fluidized and the laser spectra reveal an interesting and unreported dynamics. In Fig. 4, we show the dynamical evolution of the laser peak signal *y* at different frequencies (similar results are obtained when measuring the spectral width, not reported). The laser spectra allow to distinguish two distinct phases of the granular: the first regime, observed for 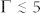 (*f* < 70 Hz in Fig. 4), is a solid phase (Fig. 1(b)) where the beads follow the motion of the plate but do not change their relative positions. The second regime, activated when 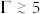 (*f* ≥ 70 Hz), is an oscillating-phase (Fig. 1(c)) in which the beads move in the whole volume of the container and site exchanges between beads take place. In the latter phase, the pronounced density fluctuations trigger the laser above and below threshold, periodically. As a result, the spectra alternate between fluorescence and random laser. We call this oscillating state a liquid-like phase. To further investigate this effect, we analyze the spectral information in the dynamics of the oscillating response of Fig. 4. We perform a Fourier-domain analysis of the temporal signals in Fig. 4, reported in Fig. 5. We then extract the fundamental frequency of oscillation by interpolating the data shifted by the temporal mean value with a single sinusoidal function. The analysis reveals two phases with well-separate solid-like and liquid-like areas; the phase transition is evidenced when the amplitude of the fit is plotted as a function of the frequency (Fig. 6). There is evidence of a resonant peak at *f* = 70 Hz in the oscillating region. The granular response is hence not flat but shows an internal structure with preferential mechanical modes. In the inset, we report the fitted frequency of the oscillating phase versus the driven frequency. The interpolated frequencies are two orders of magnitude smaller than known typical granular oscillations frequencies, among which the so-called “bouncing-bed” regime^5^. The latter is an oscillation of the whole granular like a solid, which, in dry samples, has a characteristic value *f_bb_* given by *f_bb_* = *f*/2.

To exclude subsampling and stroboscopic effects, we performed acquisition of spectra at different sampling times. We did not find relevant changes. The fact that the measured frequency is so much lower than *f_bb_* in dry granulars can be ascribed to the hosting liquid. The physics of liquid-immersed granulars is in many respects unknown, the analysis of the dynamic granular laser emission indicates the presence of very low frequency vibrational modes.

**Figure 7:**
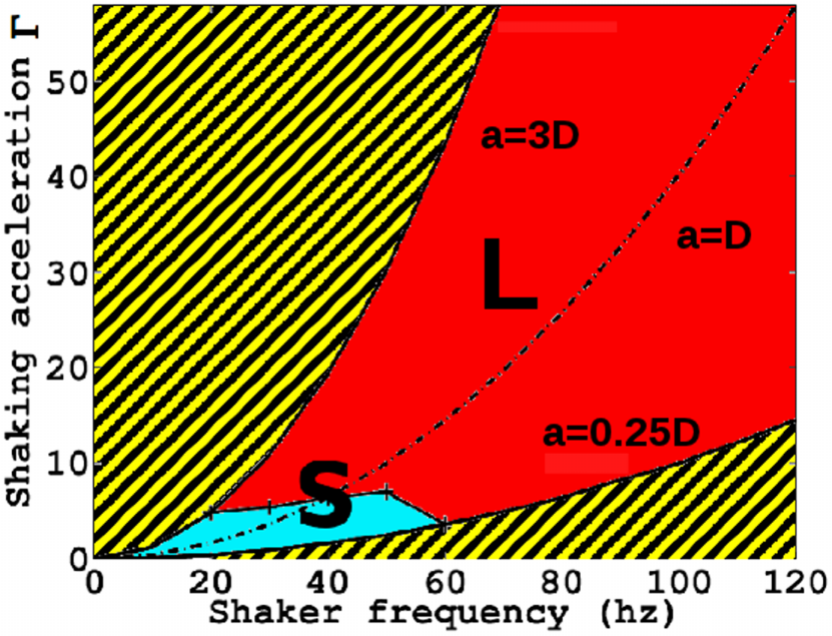
Phase diagram of glass bead with *D* = 1.0 mm with Rhodamine (B). The region in red (dark gray) corresponds to the liquid-like (G) phase, related to the laser emission spectra oscillations, the region in blue (light gray) indicates the solid-like (S) phase. The region filled with stripes represents areas out of the range of linearity of shaker response, and hence not explorable.

To better address the liquid-like state and the origin of the laser oscillations, we perform further investigations and measure a phase-diagram (see Fig. 7) to characterize the features of our sample. The adopted order parameter is the peak 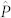 of the spectral analysis in Fig. 5. We identify the liquid-like phase to the regime in which the peak amplitude 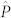 is about three times greater than the background and correspondingly, the solid-like phase when no relevant peaks are observed. We see that the liquid-like state (oscillatory dynamics) is present also below the line at which the plate displacement equals the diameter of the beads (*a* ≅ *D*), and the oscillations of the laser seem to result from the density modulation of the granular, and to depend on the grain inertia further enhanced by the presence of the liquid active medium (dyed methanol) that slows down the beads.

## Discussion

In^15^, we had shown the configurational dependence of laser emission from the shaken granular. In this work, we follow dynamically this dependence to extract informations about the structural phases of the grains. We have found evidence of a periodic time dependence of laser emission from a granular sample when it is opportunely shaken. This temporal correlation is related in a non-trivial manner to the dynamical phases of the granular. These oscillations are not related to some convective motion of a few glass particles that, absorbing light, are carried periodically through the laser spot. In fact, a setup like that we used, with an aspect ratio of 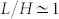 and with 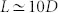, can not support granular convection rolls or undulations. We stress that a rigorous understanding of the observed phenomenon is still missing but we believe that the granular laser oscillations have to be related to a sort of pulsation in the configurational statistical properties of the out-of-equilibrium liquid granular system. In conclusion, we first provide the experimental evidence that the laser emission furnishes information on the dynamics of the granular system. Not only we distinguish two granular phases, the static and the dynamical regimes, but also provide evidence that laser analysis encodes the spectral structure of the granular collective motion. Random lasing hence provides a quantitative tool to investigate collective motion in granular matter.

Developments include applying these results to study granular systems with more complex phase-diagram and link with statistical mechanics. Additionally, the rich and collective behavior of granular matter may allow to implement a new class of random laser, tunable by the mechanical control.

## Methods

### Sample

Our sample consists of about *N* = 1.194 grams (about 1200 particles) of *D* = 1 mm diameter spherical dielectric spheres. The grains are immersed in a solution of Methanol and Rhodamine B (concentration 1 mM, fluorescence peak around 590 nm), and are contained in a square cuvette of side length *L* = 10 mm = 10*D*. The sample height in the vertical direction is 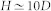 in order to have an aspect ratio 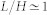. We choose this regime to reduce the variety of supported granular phases which become more complex for *L*/*H* ≫ 1^5^,^18^.

### Setup

The container is placed on a shaker that can vertically vibrate. The applying force controlling the shaker is sinusoidal at frequency *f*. The vertical displacement *a* of the granular cuvette has been measured by employing a piezoelectric accelerometer. A pump laser beam, a Q-switched 532-nm Nd:YAG laser (10 Hz repetition rate, 7 ns pulse duration and spot size 6 mm), is used, and the spot position *z* with respect to the cuvette bottom is set by a vertical motorized 25 mm translational stage. Spectral emission is measured by an electrically cooled CCD array detector (operating temperature –70 degree Celsius).

## Author Contributions

C.C., N.G., V.F. designed research, V.F. performed experiments, analyzed data and wrote the manuscript, C.C., N.G., A.P. and L.L. critically reviewed it. All authors gave their approval for the final version of the paper.
